# Evaluating the evolutionary mechanisms maintaining alternative mating strategies in a simulated bull trout (*Salvelinus confluentus*) population

**DOI:** 10.1002/ece3.9965

**Published:** 2023-04-07

**Authors:** Thaïs A. Bernos, Sarah L. Chang, Rachael M. Giglio, Kaeli Davenport, Jeff Fisher, Erin Lowery, Andrew Bearlin, Ryan Simmons, Marie‐Josée Fortin, Casey C. Day, Erin L. Landguth

**Affiliations:** ^1^ Department of Ecology and Evolutionary Biology University of Toronto Toronto Ontario Canada; ^2^ Department of Biological Sciences University of Toronto Scarborough Toronto Ontario Canada; ^3^ Department of Biology University of British Columbia Okanagan Kelowna British Columbia Canada; ^4^ Department of Ecology, Evolution, and Organismal Biology Ohio State University Columbus Ohio USA; ^5^ United States Department of Agriculture National Wildlife Research Center Ottawa Ontario USA; ^6^ Department of Wildlife Biology University of Montana Missoula Montana USA; ^7^ Seattle City Light Seattle Washington USA; ^8^ School of Public and Community Health Sciences University of Montana Missoula Montana USA

**Keywords:** alternative mating tactics, boosted regression trees, CDMetaPOP, global sensitivity approach, individual‐based spatially explicit model, riverscape genetics, salmonid, simulation, Skagit River, sneakers

## Abstract

The coexistence of distinct alternative mating strategies (AMS) is often explained by mechanisms involving trade‐offs between reproductive traits and lifetime fitness; yet their relative importance remains poorly understood. Here, we used an established individual‐based, spatially explicit model to simulate bull trout (*Salvelinus confluentus*) in the Skagit River (Washington, USA) and investigated the influence of female mating preference, sneaker‐specific mortality, and variation in age‐at‐maturity on AMS persistence using global sensitivity analyses and boosted regression trees. We assumed that two genetically fixed AMS coexisted within the population: sneaker males (characterized by younger age‐at‐maturity, greater AMS‐specific mortality, and lower reproductive fitness) and territorial males. After 300 years, variation in relative sneaker success in the system was explained by sneaker males' reproductive fitness (72%) and, to a lesser extent, the length of their reproductive lifespan (21%) and their proportion in the initial population (8%). However, under a wide range of parameter values, our simulated scenarios predicted the extinction of territorial males or their persistence in small, declining populations. Although these results do not resolve the coexistence of AMS in salmonids, they reinforce the importance of mechanisms reducing sneaker's lifetime reproductive success in favoring AMS coexistence within salmonid populations but also limit the prediction that, without any other selective mechanisms at play, strong female preference for mating with territorial males and differences in reproductive lifespan allow the stable coexistence of distinct AMS.

## Graphical abstract

We used CDMetaPOP to simulate bull trout alternative mating strategies and found that variation in sneaker success in the system was explained by reproductive fitness (72%), the length of their reproductive lifespan (21%), and their proportion in the initial population (8%).

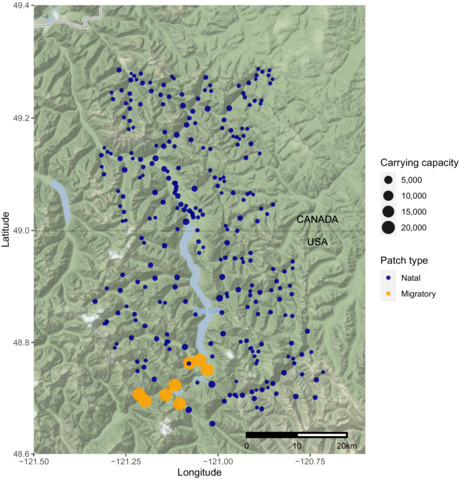

## 1 INTRODUCTION

Alternative mating strategies (AMSs), defined as differences in mating strategies within sexes, can occur in species where competition for access to breeding resources is high (Myers et al., 1986; Rubenstein, 1980). AMS can be fixed or plastic over an individual's lifetime and typically involves male‐specific discontinuities in phenotypic, behavioral, and life‐history traits (Taborsky, 1994; Taborsky et al., 2008). Male AMSs often conform to the “territorial‐sneaker” paradigm, with “territorial males” actively guarding females and defending territories, whereas “sneakers” exploit the breeding resources defended by others (Taborsky & Brockmann, 2010). Understanding the conditions under AMS persistence in the wild and the factors involved in their regulation is relevant to wildlife management, as within‐population variation in life‐history traits is important for the conservation of genetic variation in wild populations (Garcia‐Vasquez et al., 2001; Perrier et al., 2014).

Negative frequency‐dependent selection, which occurs when AMSs have a high fitness when uncommon and a low fitness when common (Wright, 1939), has been proposed as an important mechanism underlying the stable coexistence of AMS in natural populations (Gross, 1996; Myers et al., 1986). For example, if territorial males are very abundant in a population, sneakers should experience little competition from other sneaker males and have many breeding resources to exploit (Reichard et al., 2004). If negative frequency‐dependent selection operates on both morphs, it should lead to the stable coexistence of distinct AMS near an equilibrium frequency (Hutchings & Myers, 1994; Takahashi et al., 2010). However, negative frequency‐dependent selection has rarely been tested and confirmed empirically for AMS within sexes (but see Gross, 1996; Berejikian et al., 2010). Furthermore, the high amount of among‐population variation in the incidence of sneakers relative to territorial males observed in natural populations suggests that the point at which the reproductive fitness of the two strategies is equal varies among populations (Dalley et al., 1983; Hutchings & Myers, 1994; Myers et al., 1986).

Although negative frequency‐dependent selection may be a powerful mechanism maintaining genetic diversity in natural populations (Clarke, 1979), other evolutionary processes have also been invoked to explain the coexistence of several AMS within natural populations (Engqvist & Taborsky, 2016; Piche et al., 2008; Plaistow et al., 2004; Rios‐Cardenas et al., 2007; Sinervo & Lively, 1996). An obvious one is density‐dependent selection related to breeding resources, which occurs when there are negative trade‐offs between the fitness benefits of an AMS and intrasexual competition. For this mechanism to maintain distinct AMS, males with life‐history traits typically associated with the sneaker strategy must have a decreased ability to cope with greater intrasexual competition at higher population densities (Wright et al., 2020). In addition, distinct AMS can be maintained in a population when the fitness advantages of early age‐at‐maturity are balanced by differential mortality rates (Maynard Smith, 1982; Ryan et al., 1992). For example, in populations harboring fixed sneaker‐territorial AMS, sneakers may invade the population when mortality due to precocious maturation is low (Myers et al., 1986). Territorial traits can also confer lower adult mortality to males later in life, thereby resulting in equal lifetime reproductive payoffs between the two AMSs (Caswell et al., 1984). These potential mechanisms contributing to the occurrence of AMS are not mutually exclusive, and additional factors could be involved.

Simulation frameworks have proven useful to explore factors influencing the coexistence of multiple AMS that would be difficult to isolate in natural observations, such as competition (Alonzo & Calsbeek, 2010), genetic architecture (Moulherat et al., 2017), environmental conditions (Engqvist & Taborsky, 2016), and frequency‐dependent selection (Svensson et al., 2016). While these models have greatly contributed to our theoretical understanding of AMS, they have not been leveraged to investigate the mechanisms contributing to maintaining AMS in specific animal populations or ecosystems. Increased realism, however, is essential to inform the practical management of AMS within natural populations (DeAngelis & Grimm, 2014). In particular, considerations related to AMS are often incorporated in fishery management practices and policies; in endangered species, maintaining within‐population diversity residing in distinct AMS can be important for populations' long‐term persistence in changing environments (Waples & Lindley, 2018). In other management scenarios (e.g., hatchery supplementations), AMS can be considered undesirable when they decrease management performance (Zimmerman, 2003). Individual‐based simulation models can, therefore, provide an understanding of how individual‐level mechanisms (e.g., interactions between individuals and the environment) might produce patterns that are observed and can be managed at the population‐level, such as the coexistence of distinct AMS.

In this study, we leveraged the power of a spatially explicit, individual‐based, eco‐evolutionary model, Cost–Distance Meta‐POPulation (CDMetaPOP; Landguth et al., 2017), to explore the influence of several factors on the maintenance of AMS. We developed simulations reflecting AMS observed in a bull trout (*Salvelinus confluentus*) population of the Skagit River in Washington, USA. Many salmonid species, including bull trout (Baxter, 1997; Kitano et al., 2011), exhibit two distinct mating strategies: territorial males actively court and control access to females, while sneaker males exploit the breeding resources defended by territorial males (Blanchfield & Ridgway, 1999; Esteve, 2005; Gross, 1996; Maekawa et al., 1994; Maekawa & Hino, 1986). In bull trout and most other salmonids, sneaker males are typically characterized by a younger age‐at‐maturity and a smaller size than territorial males (Kitano et al., 2011; Paez et al., 2011). As sneaker and territorial males irreversibly start following divergent developmental and growth trajectories early in their lifetime, these mating strategies are commonly fixed (Aubin‐Horth & Dodson, 2004). Accordingly, there is accumulating evidence for a genetic component to traits underlying salmonid AMS, including sexual development and body size (Lepais et al., 2017; Mobley et al., 2021; Theriault et al., 2007). As CDMetaPOP allows for the consideration of individual attributes (i.e., growth, mortality), social interactions (i.e., mating, competition), and spatially explicit dispersal, it is well suited to model complex population dynamics in a realistic manner where such parameters can be empirically derived. While bull trout spawning behaviors remain vastly understudied, a broad range of salmonid AMS have been described in the literature (e.g., Baxter, 1997; Blanchfield et al., 2003; Fleming, 1996; Foote et al., 1997; Garcia‐Vasquez et al., 2001), allowing us to capture generalities underlying the coexistence of AMS in salmonid populations.

Here, we simulated bull trout established in the upper Skagit River core recovery area assuming that sneaker and territorial males coexist within the initial starting population. Our objective was to investigate factors that can affect the maintenance of sneaker males in a population. To do so, we employed global sensitivity analysis testing to examine the effects of breeding fitness, differences between sneakers and territorial males in age‐at‐maturation and reproductive lifetime length, and initial proportion of sneaker males in the population, all evaluated with metrics of relative sneaker success. The coexistence of distinct AMS is often assumed to result from negative frequency‐dependent selection, though this is largely a theoretical framework where selection favors relatively rare phenotypes, with rare empirical examples (but see Berejikian et al., 2010). As a result, our simulation study is purposely focused on other causative processes, including density‐dependent mechanisms and trade‐offs between fitness traits. We developed our models under the assumptions that: (1) territorial males are fixed in this behavior throughout their lifespan (Engqvist & Taborsky, 2016), (2) they tend to be preferred by breeding salmonid females (Auld et al., 2019; Berejikian et al., 2000; Egeland et al., 2016; Neff et al., 2008), (3) they are associated with greater sperm production, intersexual, and intrasexual competitive abilities, culminating in an overall greater breeding fitness (Bolgan et al., 2017; Dougherty et al., 2022; Koch & Narum, 2021; Kustra & Alonzo, 2020; Lehnert et al., 2017), and (4) they may also have longer lifespans since they are less susceptible to predation, cannibalism, and starvation than smaller individuals (Miller et al., 1988; Sogard, 1997; van den Berghe & Gross, 1986; but see Carlson et al., 2004). With these considerations, our model is an initial investigation of strategies related to AMS, and our findings on the maintenance of AMS can be applicable to other salmonid species and inform further simulation and empirical study of this complex life‐history behavior.

## 2 METHODS

### 2.1 Study system

**FIGURE 1:**
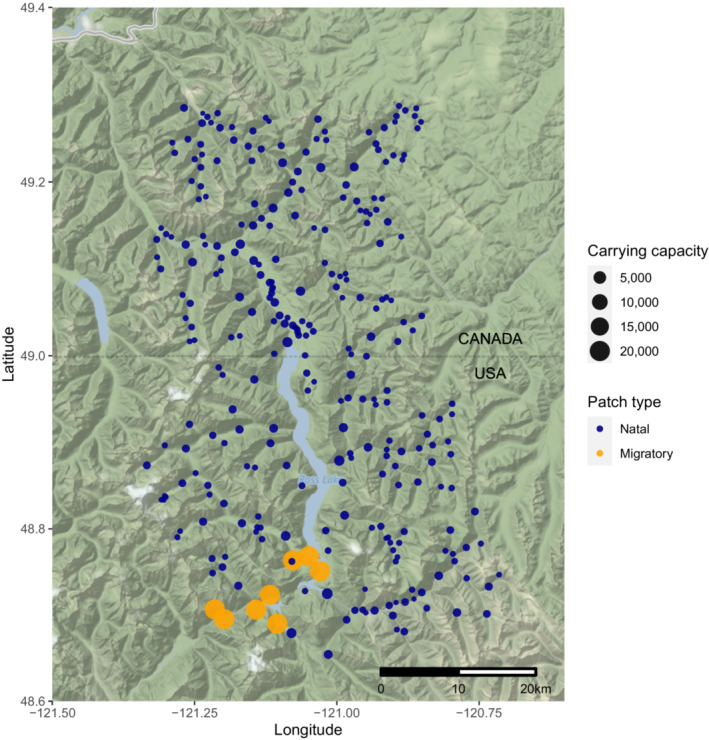
Map of the upper Skagit River system. Points mark the locations of patches used in the CDMetaPOP model. The size of the points indicates the carrying capacity of each patch, and the color of the points represents whether the patch is used for migration by bull trout within the riverine system (blue for natal patches and orange for migratory patches).

**FIGURE 2:**
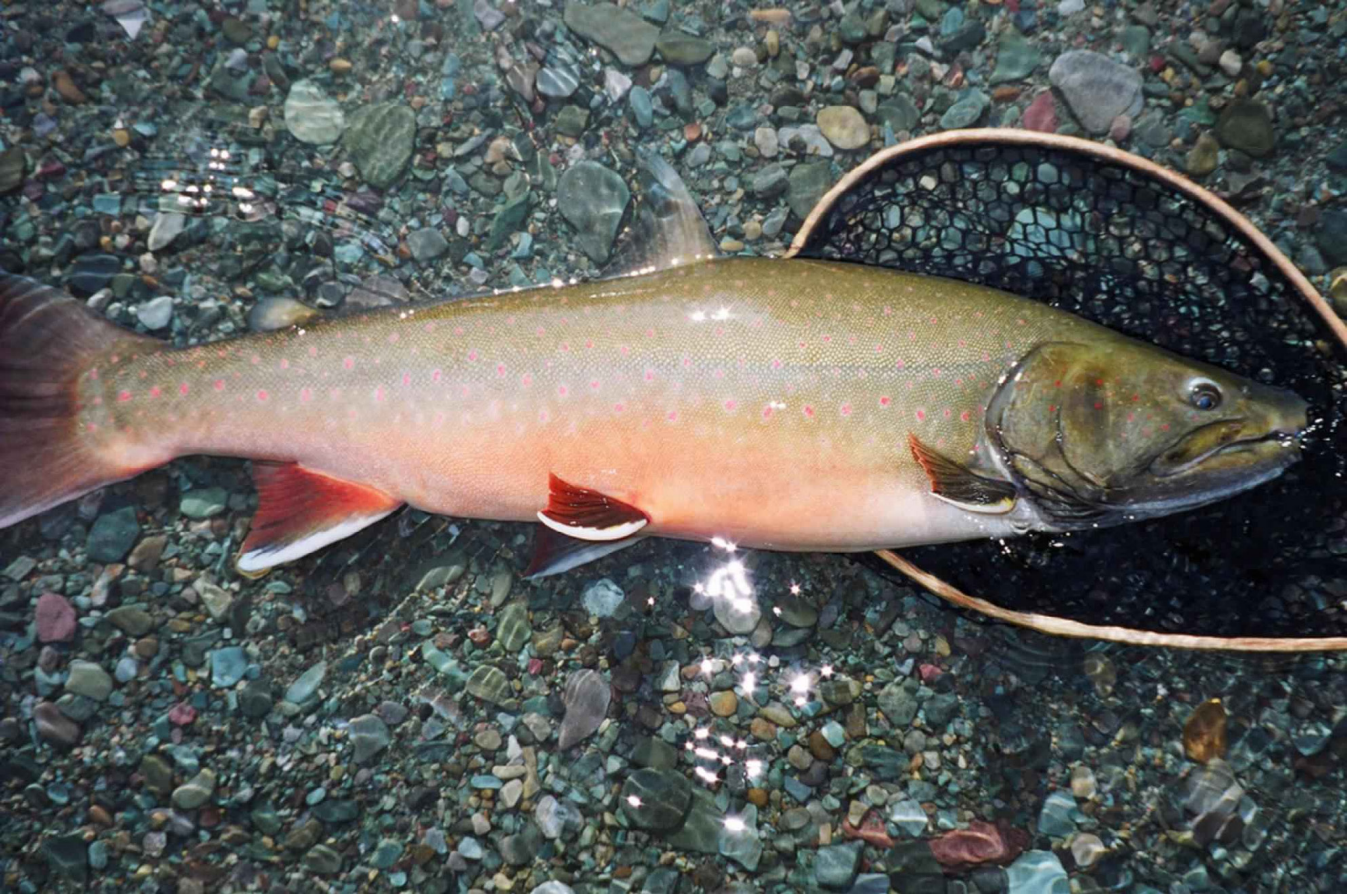
Bull trout from the Skagit River system.

The Skagit River system (Figure 1) represents the largest river feeding Puget Sound and meanders over 158.5 km through Washington state (the United States, US) and British Columbia (Canada), and it contains half of the bull trout populations of the Puget Sound area (Zimmerman & Kinsel, 2010). We specifically focused on the upper Skagit, a core area for bull trout recovery, with upper (above Gorge dam) and lower (mainstem and tributaries below Gorge) recognized as distinct core areas in the Coastal Recovery Unit of the USFWS bull trout recovery plan (US Fish and Wildlife Service, 2015). The watershed supports diverse Bull Trout (Figure 2) life histories, including mig in the lower portion of the upper Skagit system and migrate back to their natal headwater streams to spawn (see Figure 1) and resident individuals who spend most of their lives in streams.

## 3 SIMULATION MODEL

To simulate bull trout sneaker dynamics, we used the modeling software CDMetaPOP v2.33 (Landguth et al., 2017). CDMetaPOP is a demogenetic modeling framework simulating the movement of individuals across heterogeneous landscapes composed of “habitat patches.” Within each patch, population growth is influenced by patch carrying capacity, itself a density‐dependent function of patch size and estimated fish density (one individual per 100 m^2^; see Nathan et al., 2019).

### 3.1 Alternative mating strategies

Here, we incorporated two distinct AMSs to the population: sneaker and territorial strategies, with contrasting age‐at‐first‐maturity and mortality‐by‐age for males. Within the CDMetaPOP framework, AMS‐specific phenotypic traits were linked to individuals by genotype. We focused on the simplest case of genetic polymorphism involving a single locus with a dominant and a recessive allele: recessive homozygote individuals (L0A1 = 2) followed the developmental trajectory of the sneaker AMS, whereas individuals that were heterozygous (L0A1 = 1) or homozygous for the dominant allele (L0A1 = 0) followed that of territorial males. While we assumed here that the AMS coexisted in the population at the beginning of each simulation, we varied the relative proportion of sneakers to territorial individuals (see Table 1, Table S1).

**TABLE 1:** Parameters varied across simulated scenarios of alternative mating strategies for bull trout in the upper Skagit River system.

Latin hypercube parameters	Continuous range	Explanation
Assortative mating	1–3	*c* = 1 is equivalent to random mating, *c* > 1 indicates higher female preference for territorial males
Sneaker maturation curve slope	0.01704–0.05112	Increasing slope indicates earlier maturity at smaller size classes
Sneaker male proportion	2%–50%	Proportion of males expressing sneaker phenotype at model initialization per patch
Sneaker size mortality back %	3.75%–60%	Size class specific mortality percentages applied in natal patches after immigration

### 3.2 Breeding fitness

Female salmonids generally prefer males of larger size, typically correlated with increased fecundity, over smaller males (Auld et al., 2019; Koch & Narum, 2021). In the CDMetaPOP framework, this was best captured by a “Dominant‐Preference” model (Day et al., 2020; M'Gonigle & Fitzjohn, 2009). Under this model, the probability that a female (*ψ*_*i,j*_) will mate with a territorial male is given by the following equation:

(1) $$\psi_{i,j}=c^{\delta_{i,j}}\;f_{j}$$

here, *c* is the strength of female's mating preferences (assortative mating factor), *f*_*j*_ is the frequencies of each AMS strategy in the female's sample of males, and *δ*_*i,j*_ is the Kronecker's Delta (this is equal to 0 when genotypes of the female and the male were different, and equal to 1 when genotypes are the same). *Ψ*_*i,j*_ can be interpreted as the result of female mate preference mechanisms, including premating (e.g., mate preference) and cryptic (e.g., sperm selection) choices (M'Gonigle & Fitzjohn, 2009). As implemented in CDMetaPOP, females of any genotype mated with all territorial males with equal probability and were less likely to mate with sneaker males when *c* > 1. For example, when *c* = 2 in a patch with equal frequencies of sneaker males and territorial males, females were twice as likely to mate with a territorial male than a sneaker male.

At each time step (defined as a year), females preferentially selected males for breeding (polyandry specified through female and male replacement) with equal clutch sizes across breeding pairs with multiple male breeding events, where fathers and resulting genes are randomly selected to generate offspring based on a Poisson draw of the mean fecundity value derived as a function of the length of the female (number of eggs = 9.5576 × exp(Length × 0.0181)) (Bowerman, 2013). After reproduction events, AMS‐specific genotypes were assigned to both female and male offspring generated by Mendelian inheritance to allow the trait to pass on through multiple generations, with AMS‐specific phenotypic traits only expressed in males. This new population subsequently includes surviving individuals, inclusive of both newly produced offspring and the survivors from previous time steps. Survival probability depends on size (or age), which affects fecundity and growth.

### 3.3 Size‐dependent maturity and sneaker‐specific mortality

On average, bull trout has an estimated generation time of 4–7 years (Mogen & Kaeding, 2005; Roth et al., 2021). Generally, sneaker males are characterized by an earlier age‐at‐maturation than territorial males. Smaller individuals can have a higher mortality than larger individuals due to their higher susceptibility to predation (Sogard, 1997), cannibalism (Klemetsen et al., 2003), and starvation (Miller et al., 1988). In CDMetaPOP, growth rate typically declines as individuals become older and larger, which is captured in the Von Bertalanffy equation (Von Bertalanffy, 1957). The probability of maturation was then modeled as a function of size (Downs et al., 1997):

(2) $$P\left(\text{mature}\right)=\exp\left(A+B\times \text{Length}\right)/\left(1+\exp\left(A+B\times \text{Length}\right)\right)$$

where *A* and *B* are fit based on individual length. For females, *P*(mature) was fixed based on empirical data and is not affected by AMS‐specific phenotype. For sneaker males, the probability associated with maturing at a younger age, and thus smaller size, was greater than for territorial males.

Mortality operated at the habitat‐patch level and the AMS‐class level for all individuals. Mortality at the habitat‐patch level reflects density‐dependent mortality within age/size classes. We modeled an additional mortality linked to the sneaker strategy in males that varied by scenario (Table 1) and was applied across all age classes during the yearly life cycle. Additionally, immigration and emigration processes within the model result in density‐independent mortality simulating migration survival.

Individuals migrated to overwintering habitats according to an inverse linear dispersal kernel based on geographic distances between habitat patches (e.g., Nathan et al., 2019). Individuals generally returned to their natal habitat for spawning; however, straying could occur at low probabilities. After returning to their spawning habitat, local dispersal among spawning habitat patches was controlled based on a fitted leptokurtic dispersal kernel allowing both short‐ and long‐distance dispersal (Radinger & Wolter, 2013). Meta‐analyses revealed that dispersal distances can be estimated by fish length, stream size, and aspect ratio of the caudal fin (Radinger & Wolter, 2013). Here, mean dispersal distances for the two components (1.79 km and 3.49 km, respectively) were predicted using the R package “fishmove” (Radinger & Wolter, 2013) (average fish size = 191 mm; stream order = default value, 6; aspect ratio of the caudal fin = 1.87) to accurately reflect bull trout movement and implemented into the simulation model with the FIDIMO movement function.

## 4 ANALYSES

### 4.1 Baseline scenario and simulations

**FIGURE 3:**
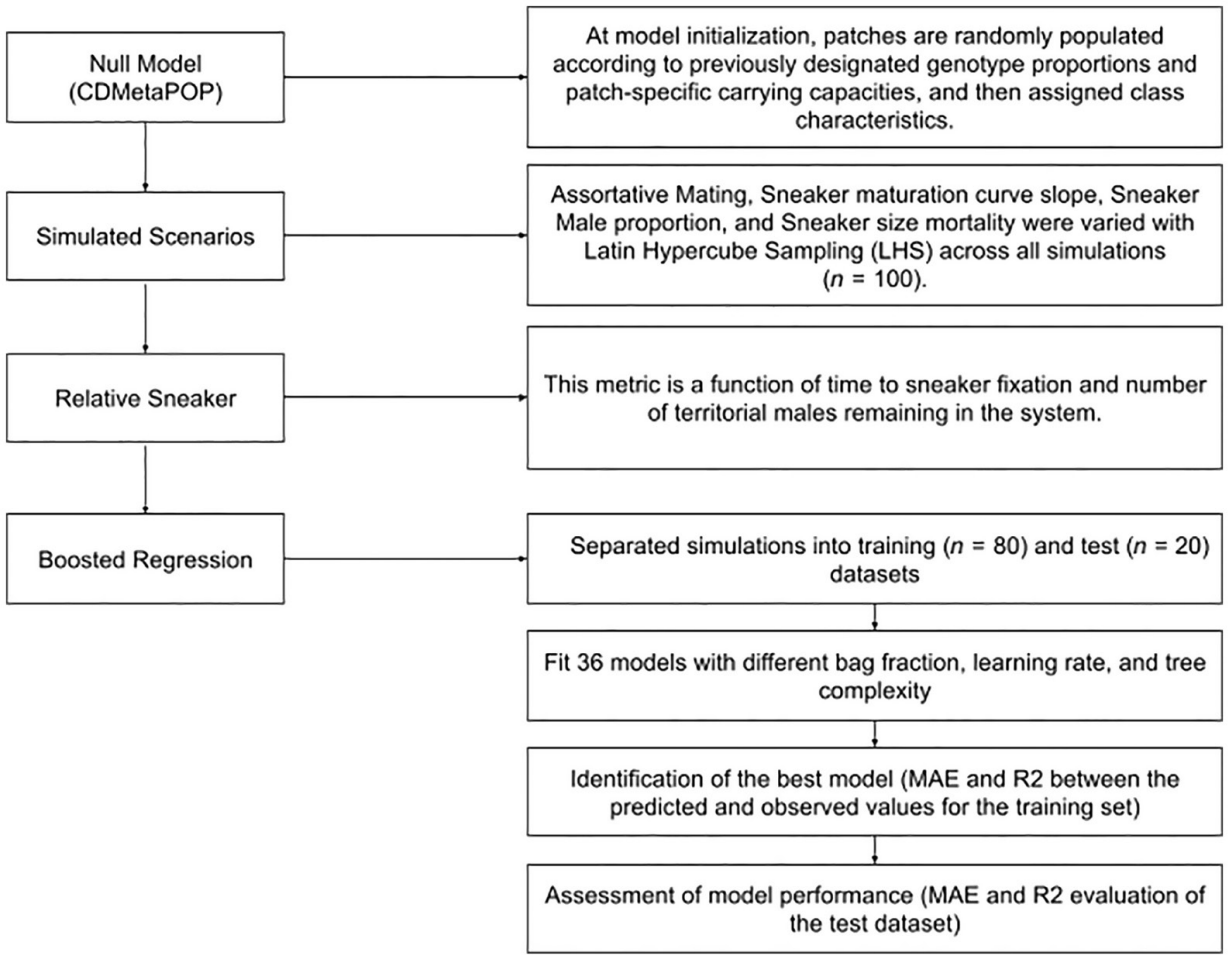
Flowchart of analytical methods used to evaluate the effect of selective mechanisms on the persistence of alternative mating strategies in a simulated population of bull trout (*Salvelinus confluentus*) in the Skagit River.

We first created a null scenario as a baseline scenario to compare to alternative parameter combinations and analyses (Figure 3). In this null scenario, there were no phenotypic distinctions between the two AMSs (they followed the same growth and maturity trajectories), there were no differences in mortality, and the model was initialized with equal proportions of sneaker and territorial genotypes. Females randomly mated with males of both sneakers and territorial genotypes. Migration was set as an inverse linear transformation of a cost–distance matrix to ensure movement of individuals between migration and spawning grounds. We ran this null model for 10 iterations with an initial 50‐year burn‐in for the genetics and the demographics followed by 300 years, and we outputted individual files at 10‐year intervals (see Table S1 for a list of fixed and variable parameters). We ensured that this baseline scenario emulated some of the processes observed in the real system, including a stable proportion of inhabited patches over time and identical age class structures.

### 4.2 Global sensitivity analysis

Global sensitivity analysis approaches allowed us to vary all the parameters of the simulation simultaneously, which can help identify potential interactions among parameters and non‐linear responses and evaluate how uncertainties in parameter values affect the outcome of the model (Wainwright et al., 2014). To explore the effects of mating preference, maturation, sneaker male proportion, and mortality on sneaker dynamics in this system, we simulated 100 scenarios with different parameter combinations as a simplified representation of negative frequency‐dependent selection. Specifically, we varied the following parameters: assortative mating factor [range = 1–3], maturation slope [0.01704–0.05112], starting sneaker male proportion [2–50%], and additive mortality to sneaker males [3.75–60%] (Table 1). To effectively sample the parameter space (i.e., parameter values within the aforementioned ranges for four parameters), we used the Latin Hypercube Sampling (LHS) method implemented in the R package “lhs” (Carnell, 2022). Latin Hypercube Sampling divides the parameter space into subdivisions of equal sizes and then draws samples randomly within these subdivisions to produce orthogonal parameter sets. By doing so, it maximizes diversity in the combination of parameters configurations (see parameter space in Figure S1), thereby allowing for more efficient inferences.

### 4.3 Metric of relative sneaker success

To compare the relative success of sneaker males among simulated scenarios, we designed a metric of relative sneaker success (adapted from Day et al., 2020). The relative sneaker success metric combined time to sneaker fixation and, when it was applicable, the number of territorial males observed in the population at the end of the simulation. It was computed as follows:

(3) $$\text{Relative sneaker success}=1-\frac{\left(\frac{T_{\mathrm{fix}}-T_{\max}}{T_{\max}-T_{\min}}+\frac{N_{\mathrm{ter}}-N_{\min}}{N_{\max}}\right)}{2}\;,$$

where *T*_fix_ is the time for sneaker fixation, *T*_min_ and *T*_max_ are the minimum and maximum times to sneaker fixation across the 100 simulation scenarios, respectively, *N*_ter_ is the observed territorial males in the final year of the simulation, and *N*_min_ and *N*_max_ are the minimum and maximum number of territorial males in the final year across the 100 simulation scenarios, respectively. In cases where a simulation did not become fixed for sneakers, *T*_fix_ was equal to *T*_max_ (300 years); when there were no territorial males left at the end, *N*_ter_ was set to 0. Thus, our metric of relative sneaker success was bounded between 0 (lowest sneaker success) and 1 (highest sneaker success).

### 4.4 Boosted regression trees

To analyze the relationship between relative sneaker success and predictor variables (Table 1), we fitted boosted regression trees (BRTs) using the R package “dismo” (Hijmans et al., 2017). BRTs are a powerful statistical method because they combine boosting (an algorithm to build and combine multiple models into more accurate aggregated prediction) across a large number of regression trees (models partitioning the data into subgroups with similar response values using binary splits) (Elith et al., 2008). BRTs were ideal for our analysis because this non‐parametric approach can be used to fit non‐linear relationships and automatically handles interactions among predictor variables.

To fit BRTs, we first randomly separated our dataset into a training (70 simulations) and a testing set (30 simulations). In the BRT framework, four parameters need to be defined: the bag fraction (proportion of the training set used for each fit), the learning rate (contribution of each tree to the model), the tree complexity (number of nodes in the tree), and the number of trees (determined by the interaction between tree complexity and learning rate) (Elith et al., 2008). To identify the optimal parameter combination to model the metric of relative sneaker success, we then fitted 36 BRT models spanning a range of parameter values (bag fraction = 0.5, 0.6, 0.7; tree complexity = 2, 3, 4; and learning rate = 0.01, 0.005, 0.001, 0.0005) to the training dataset. We assumed a Gaussian error distribution. The optimal model, defined as the one producing the lowest mean absolute error, used a bag fraction = 0.5, tree complexity = 2, and a learning rate = 0.01. We then removed non‐informative predictors using backward variable elimination. To assess the performance of the final model, we calculated the validation *R* ^2^; that is, the proportion of the variance in the testing set that was correctly predicted by our final model. To do so, we used a Spearman correlation to evaluate the strength of the correlation and the mean absolute errors (MAE) between predicted and observed values for the testing set.

## 5 RESULTS

### 5.1 Baseline and simulated scenarios

**FIGURE 4:**
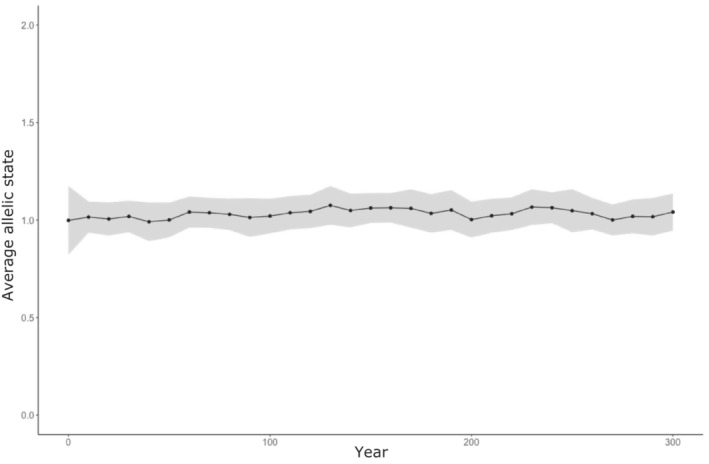
Average allelic state of all individuals across all patches in the baseline scenario. Recessive homozygote individuals (L0A1 = 2) followed the developmental trajectory of the sneaker AMS, whereas individuals that were heterozygous (L0A1 = 1) or homozygous for the dominant allele (L0A1 = 0) follow that of territorial males.

In the baseline scenario, with no differences in life‐history traits between sneaker and territorial males, allelic frequencies plateaued around one (Figure 4). Initially, the system included a mix of sneaker (0) and territorial males (1 and 2); however, after 10 years, most individuals remaining in the system were heterozygous territorial males. In contrast, sneaker males became the dominant phenotype in most simulated scenarios; as a result, the metric of relative sneaker success was centered around 0.66 [range = 0–1]. For example, in 71 of the 100 simulated scenarios, sneaker males became fixed before the final year. Mean time to sneaker fixation was 223 years [range = 152–298]. In scenarios where sneaker males were not fixed, the mean number of territorial males remaining in the final year of the simulation was 16,292; it ranged from 57 (<0.001% of the male population) to 85,095 (62% of the male population). None of the simulations resulted in the fixation of territorial males, and there was only one simulation where territorial males were more abundant than sneaker males after 300 years.

### 5.2 Boosted regression trees

**FIGURE 5:**
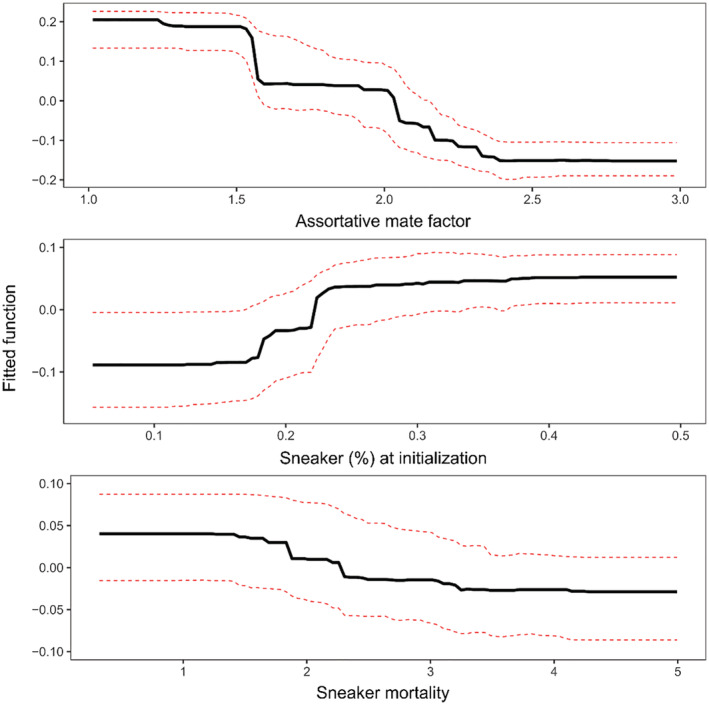
Assortative mate factor, proportion of sneaker at initialization, and sneaker mortality explained, respectively, 71.6%, 20.6%, and 7.8% of the summed variance importance across the 1000 bootstrapped trees on the metric of relative sneaker success in simulated bull trout populations. The partial response plots are calculated for each predictor variables while averaging the others. The solid black line represents the mean prediction averaged across 1000 bootstrapped samples, and the red dotted lines represent the minimum and maximum fitted response.

We found that most of the variation in the metric of relative sneaker success was explained by assortative mating factor, sneaker proportion at initialization, and sneaker‐specific mortality (Figure 5). These explained, respectively, 71.6%, 20.6%, and 7.8% of the summed variance importance across the 1000 bootstrapped trees. By contrast, sneaker maturation curve was not a substantial contributor to the model. The final BRT model had good predictive power; a large proportion of the variance of the testing set was explained by the model derived from the training set (0.793), and accordingly, the difference (MAE = 9.7%) between observed and predicted values in the testing set was relatively small.

Partial response plots (Figure 5) showed that relative sneaker success tended to decrease with the strength of female preference for territorial males (assortative mating factor), increase with the proportion of sneaker males at initialization, and decrease with increasing sneaker‐specific mortality. For the assortative mating factor, the metric of relative sneaker success showed the steepest decline around *c* = 1.5 (i.e., when females were 1.5 times more likely to mate with territorial males than sneakers if the two AMSs were present at equal frequencies), and the strongest gradual decline between *c* = 2 and 2.5. Relative sneaker success increased the most when the proportion of sneakers in the initial population varied between ~17% and 25%. Relative sneaker success declined gradually with sneaker‐specific mortality for values between 1.5 and 3.

## 6 DISCUSSION

While AMSs are prevalent across salmonid species, we do not fully understand the mechanisms facilitating their coexistence. To evaluate the circumstances under which AMS would coexist in wild populations, we simulated an existing bull trout population in a riverscape using general assumptions applicable to many salmonid populations. We were interested in a scenario where two distinct AMSs were already present in the population, and we focused on selection mechanisms favoring their stable coexistence and persistence in the population (i.e., female mating preference, sneaker proportion in the initial starting population, sneaker‐specific mortality, and variation in age‐at‐maturity). Our simulations were able to reproduce the observed heterogeneity in AMS expression across salmonid species, where some populations are found to support multiple AMS concurrently, whereas many others are fixed for one AMS. Nonetheless, in 99% of our simulations, sneaker males either invaded the system or became the prevalent AMS. To the best of our knowledge, this was the first study of AMS grounded in a natural system, thereby providing a novel perspective on the evolutionary mechanisms contributing to the co‐occurrence of distinct AMS within salmonid populations.

While AMSs have been observed in bull trout (Baxter, 1997; Kitano et al., 2011), the species remains under‐represented in the current body of literature on AMS. Accordingly, the prevalence of sneakers relative to territorial males, their age‐at‐maturity, and lifespan length were unknown for the bull trout population of the Skagit River, as field studies failed to sample and identify sneaker males. Furthermore, the prevalence and expression of AMS vary considerably across salmonid species and populations (Dalley et al., 1983; Myers et al., 1986; Valiente et al., 2005). The value of these parameters, therefore, constitutes substantial sources of uncertainties, and the global sensitivity analysis allowed us to explore how these uncertainties affected the modeling results. One benefit of this simulation approach was that it enabled us to capture uncertainties in the parameter values reflecting our system (Prowse et al., 2016). The results of our simulations are also generalizable as they can be extended to other salmonid species or populations with AMS characteristics pertaining to the sneaker strategy.

The extent of female preference for territorial males played an important role in the maintenance of AMS in our simulations, but it was not enough to achieve the stable co‐existence of distinct AMS over 300 years. Indeed, the only apparently stable simulation was achieved when female preference for territorial males was 2.4‐fold higher than for sneaker males, and the proportion of sneaker males was initially low (5%); as it increased by 0.1% per year, sneakers would have eventually overtaken the simulation if we had run it longer. In salmonids, female preference for territorial males is well documented (reviewed in Auld et al., 2019) and known to contribute to territorial males' mating advantage over sneaker males through pre‐ and post‐fertilization behaviors (Makiguchi et al., 2016; Rosengrave et al., 2008). These include hostility toward males smaller than themselves (Baxter, 1997), delayed spawning until a larger male is in position to fertilize her eggs (Berejikian et al., 2000), and favoring the sperm of one male over another post‐spawning (Rosengrave et al., 2008; Young et al., 2013). Similar variation in female preference has been documented in a number of species and was suggested as an alternative to frequency‐dependent selection for maintaining AMS in Swordtail Fish (*Xiphophorus multilineatus*) (Morris et al., 2010; Rios‐Cardenas et al., 2007) and Ruffs (*Philomachus pugnax*) (Hugie & Lank, 1997; Widemo, 1998). In contrast, our results suggest that while it influences the coexistence of AMS, female choice alone may not be sufficient to explain the stable coexistence of distinct AMS.

In fact, for a wide range of parameter values, our models predicted that sneaker males would invade the system instead of coexisting at equilibrium with territorial males. This result was surprising as the ratio of sneaker to territorial males documented in empirical studies is highly variable across and within salmonid species (Baxter, 1997; Blanchfield et al., 2003; Gross, 1996; Myers et al., 1986; Taborsky, 1994; Valiente et al., 2005), and it remains that territorialism in males is likely an evolutionarily stable strategy in some systems. Furthermore, we did not inherently assume that, beyond early maturation, sneaker males had any other selective advantage over territorial males; instead, we attempted to counter any potential behavioral fitness advantage with a combination of severe mortality costs through sexual competition and reduced lifespan length. Still, the strongest simulation result emerged from early age‐at‐maturation and the resulting shorter generation time. This gave such a strong fitness advantage to sneakers that, even when the initial proportion of sneakers present in the system varied, territorial males were unable to become fixed in our model system. Additionally, they comprised of less than 90% of the males, their estimated prevalence across multiple salmonid species (Young et al., 2013). As opposed to other simulations where the stable existence of distinct AMS was achieved (Engqvist & Taborsky, 2016; Sinervo & Lively, 1996), we purposely chose to omit negative frequency‐dependent selection in our simulation. This parameter alone could be the mechanism behind the overwhelming prevalence of the sneaker strategy in most of our simulations. While negative frequency‐dependent selection, known as a “powerful, perhaps a dominant, factor maintaining genetic diversity” within populations (Clarke, 1979), might indeed underlie the coexistence of sneaker and territorial males in natural populations, other causative mechanisms maintaining diversity in natural populations may alternatively be involved (Brisson, 2018). These include, among others, directional selection in changing environments, sexually antagonistic selection (where an allele could be beneficial to one sex and harmful to the other), and density‐dependent fitness mechanisms (Connallon & Clark, 2014; Kisdi, 1999).

Alternatively, the prevalence of sneaker males in our simulations may highlight the importance of trade‐offs between mating success and survival for the coexistence of distinct AMS (Maynard Smith, 1982; Ryan et al., 1992). Indeed, studies previously suggested that sneaker males could invade a population when the mortality associated with early maturation was relatively low (Myers et al., 1986) or when natural selection (e.g., intense fishing pressure on territorial male) disproportionately favored the sneaker phenotype (Caswell et al., 1984). In our simulations, density‐dependent mortality reflecting competition for limited resources (e.g., space, food) was age‐dependent and equally impacted sneaker and territorial males, and although we modeled survival differences between the two morphs, the sneaker‐specific additive mortality was applied across all age classes. As a result, early maturing sneaker males might have had a consistently higher probability of mating during their lifetime than the territorial males, effectively countering the disadvantages related to their reproductive fitness. In salmonids, it is not clear which AMS has the highest probability of survival: for example, larger males may be more vulnerable to predators (Carlson et al., 2004) and fishing pressures (Caswell et al., 1984), but sneaker males may not live as long on the spawning ground (Gross, 1996) and exhibit shorter lifespans because they allocate resources to maturation rather than growth (Myers, 1984). However, according to our simulations, sneaker males' mortality rates would have to be substantially higher than those of the territorial males to achieve equal fitness without selective mechanisms maintaining polymorphisms at play.

Discussing whether these results are comparable with natural systems is compounded by several factors. First, field studies of the factors regulating the relative incidence of AMS typically focus on populations where sneaker and territorial males coexist (Rios‐Cardenas et al., 2007; Valiente et al., 2005). As a result, the prevalence of distinct AMS in natural populations is unknown and it is possible that, when AMSs are genetically fixed, their coexistence is far less frequent than the fixation of one AMS. Second, field studies are typically conducted over short temporal scales (Myers et al., 1986; Zimmerman, 2003). As a plethora of environmental factors may influence the relative proportion of sneaker to territorial males and the expression of sneaker phenotypes in the short term (Aubin‐Horth & Dodson, 2004; Paez et al., 2011), it is difficult to link those extant estimates to long‐term population fluctuations through standard monitoring techniques. These issues are further compounded by difficulties associated with counting sneaker males in the wild, as sampling techniques tend to be selectively biased for fish exhibiting traits typically associated with territorial mating strategies (e.g., fish with larger body size, greater mobility) (Bohlin et al., 1989; Rudstam et al., 1984). As a result, sneaker males' prevalence may often remain underestimated in populations where AMSs coexist (Perrier et al., 2014).

Finally, our study has limitations that could be expanded upon by future work. In particular, the stable coexistence of distinct AMS could be facilitated by mechanisms that were simplified in our simulations. We were able to simulate multiple paternity, wherein multiple males sire a clutch, which has been observed in salmonid breeding events (Berejikian et al., 2010; Garcia‐Vasquez et al., 2001; Watanabe et al., 2008), but with a simplified framework where successful males (irrespectively of their phenotype) contributed equally to the clutch, rather than reflecting greater sperm contribution from territorial males. Instead, the probability of female mating with sneaker males increased with the encounter rate of mature sneaker rate but was limited by female mating preference. While this caveat removes some of the biological realism of our simulations, it is unlikely to invalidate our conclusions; although they would have done so more slowly, sneaker males would have eventually invaded the system (Hutchings & Myers, 1994; Tentelier et al., 2016).

Furthermore, we assumed that a simple genetic architecture (one locus with two alleles, one recessive, and one dominant) gave rise to two distinct AMSs. This simplification was justified by two lines of reasoning. First, the observation that some of the important traits characterizing distinct AMS in salmonids appear to be dominated by a small number of genes with a major effect (Ayllon et al., 2015; Mobley et al., 2021; Piche et al., 2008). Second, the possibility that AMS can be modeled as a threshold trait—that is, although age‐at‐maturity is likely polygenic, whether a male matures at a given age or not is under simple genetic control (Hazel et al., 1990; Hutchings & Myers, 1994). While we assumed that sneaker alleles were recessive, we generally expect beneficial dominant alleles to increase in frequency more rapidly than beneficial recessive alleles: thus, our conclusions would hold (the incidence of sneaker males in our simulated populations would have increased even more rapidly) if the allele was dominant. Accordingly, we encourage future work to focus on how complex breeding behavior and genetic architectures might influence the mechanisms shaping the maintenance of AMS in salmonids.

In conclusion, by simulating AMS at the individual level, we were able to display that female mating preference, sneaker‐specific mortality, and variation in age‐at‐maturity affected the coexistence and relative success of sneaker and territorial males. Our simulations of AMS, which incorporated more realism and environmental parameters than previous models, indicate that one mating strategy (early maturing sneakers) will likely prevail over long time scales if there are no other mechanisms (e.g., density‐dependent selection, sneaker‐specific selective pressures) at play. Our findings may have important ramifications for management strategies aiming to conserve the diversity of mating strategies within and between salmonid populations. Indeed, the management and production of males exhibiting AMS differing in age‐at‐maturation and size have a long history in salmon management and can vary widely between fish management programs. Historically, management programs often tried to reduce the contribution of sneaker gametes to the population, as they often prioritized the economic and popular appeal of larger fish (Van Doornik et al., 2002). Recently, more emphasis has been placed on the importance of maintaining diversity in phenotypes and life histories, as it is critical for the long‐term persistence of healthy populations in rapidly changing environments. Hatcheries, therefore, follow a range of sneaker spawning guidelines, but now strive for a level of contribution proportional to their presence in the natural population. However, our results suggest that prioritizing mating events with larger males instead could favor the coexistence of sneaker and territorial males within salmonid populations (Hankin et al., 2009).

## AUTHOR CONTRIBUTIONS

**Thaïs Bernos:** Conceptualization (equal); data curation (equal); formal analysis (equal); investigation (equal); methodology (equal); project administration (equal); resources (equal); supervision (equal); validation (equal); visualization (equal); writing – original draft (equal); writing – review and editing (equal). **Sarah L. Chang:** Conceptualization (equal); data curation (equal); formal analysis (equal); investigation (equal); methodology (equal); project administration (equal); resources (equal); software (lead); supervision (equal); validation (equal); visualization (supporting); writing – original draft (equal); writing – review and editing (equal). **Rachael Marie Giglio:** Conceptualization (equal); data curation (equal); formal analysis (equal); investigation (equal); methodology (equal); project administration (equal); resources (equal); software (lead); supervision (equal); validation (equal); visualization (equal); writing – original draft (equal); writing – review and editing (equal). **Kaeli Davenport:** Conceptualization (equal); data curation (equal); investigation (equal); methodology (equal); project administration (equal); resources (equal); supervision (equal); validation (equal); visualization (equal); writing – original draft (equal); writing – review and editing (equal). **Jeff Fisher:** Resources (supporting); writing – review and editing (supporting). **Erin Lowery:** Resources (supporting); writing – review and editing (supporting). **Andrew Bearlin:** Resources (supporting); writing – review and editing (supporting). **Ryan Simmons:** Resources (supporting); writing – review and editing (supporting). **Marie‐Josee Fortin:** Conceptualization (equal); investigation (equal); methodology (supporting); writing – review and editing (supporting). **Casey C. Day:** Conceptualization (equal); investigation (equal); methodology (supporting); software (lead); writing – review and editing (supporting). **Erin L Landguth:** Conceptualization (equal); funding acquisition (lead); investigation (equal); methodology (supporting); software (lead); writing – review and editing (supporting).

## CONFLICT OF INTEREST STATEMENT

The authors declare that there are no competing interests.

### OPEN RESEARCH BADGES

This article has earned an Open Data badge for making publicly available the digitally‐shareable data necessary to reproduce the reported results. The data is available at https://github.com/Linkr1/SalvelinusConfluentus_Simulation.

## Supporting information


Appendix S1

## Data Availability

All parameter data have been deposited in DRYAD (https://doi.org/10.5061/dryad.cvdncjt7t). CDMetaPOP is available at https://github.com/ComputationalEcologyLab/CDMetaPOP. Additional scripts used for analysis and model parameterization are available at: https://github.com/Linkr1/SalvelinusConfluentus_Simulation and https://github.com/changsarahl/CDmetaPOP‐visualization.
