# Supplementary material for: Evaluating the evolutionary mechanisms maintaining alternative mating strategies in a simulated bull trout (Salvelinus confluentus) population
Source: Ecol Evol. 2023 Apr 7;13(4):e9965. doi: 10.1002/ece3.9965 (PMC10082177; doi:10.1002/ece3.9965)
Supplement: Supplementary file 1 — Appendix S1 [file ECE3-13-e9965-s001.docx]

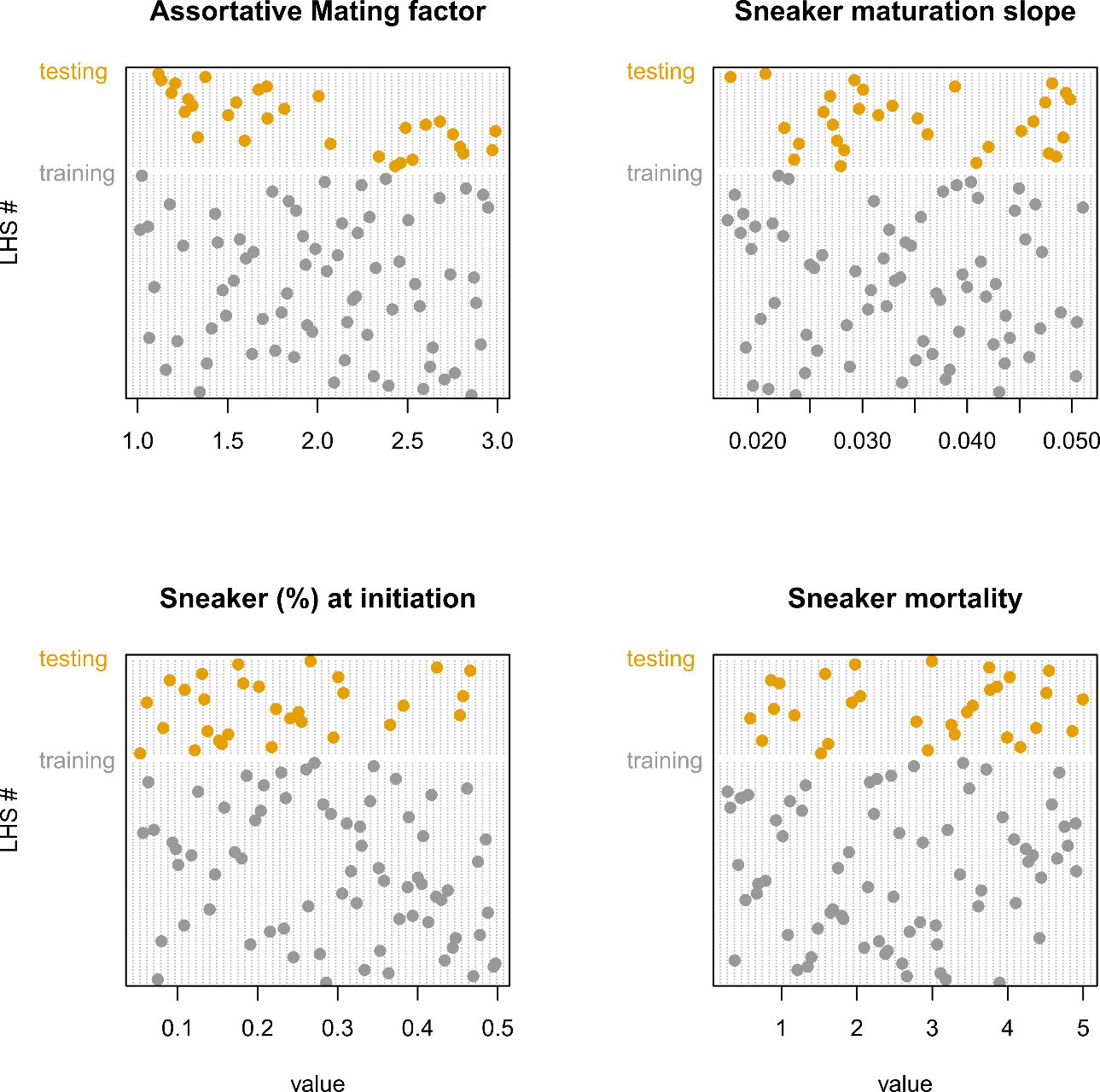


Suppl Fig. 1: Parameter combination sampled for each LHS run with Latin hypercube sampling showed for the testing (orange, N = 30) and the training (grey, N = 70) datasets used for the boosted regression tree analysis.

Suppl. Table 1: Parameter values used in the simulation model

| **Parameter** | **Value** | **References** |
| --- | --- | --- |
| ***Class Characteristics*** |  |  |
| Age Class Sizes (mm) | 75, 196, 279, 326, 351, 363, 371, 375 | Seattle City Light  (unpublished data) |
| Size Standard Deviations (mm) | 5 | Fraley and Shepard 1989;  Zymonas 2006 |
| Age Mortality | Dominant: 50% at age 7  Male Sneaker: 25% age 4, 50% age 5, 75% age 6, 80% age 7, varied by scenario | Calibrated to match empirical age distribution - Seattle City Light (unpublished data) |
| Migration | 0, 0.075, 0.283, 0.403, 0.471, 0.508, 0.540, 0.572 | Zymonas 2006; Belanger 2002 |
| Straying | Dominant: 0.01  Sneaker: 0.001 | Expert opinion |
| ***Patch Parameters*** |  |  |
| Carrying Capacity (K)      Starting Proportion (N0) | Varied by patch; mean_natal_ = 937  mean_migratory_ = 20,000      Varied by scenario | Nathan et al. 2019  Modified from Nathan et al. 2019 |
| Temperature Out    Temperature Back | 6.587    12 | Modified from Nathan et al. 2019  Modified from Nathan et al. 2019 |
| GrowDaysOut | 289 | Nathan et al. 2019 |
| GrowDaysBack | 76 | Nathan et al. 2019 |
| ***Population Parameters*** |  |  |
| Assortative Mating Factor (c) | Varied by scenario |  |
| Maturation slope; intercept (Dominant Male, all females) | 0.01704; -6.04431 | Bowerman 2013 |
| Maturation slope; intercept (Sneaker Male) | Varied by scenario |  |
| Egg_Mean__par1 | 126.07 | Al-Chokhachy and Budy 2008 (length to fecundity equation) |
| Egg_Mean_par2 | 0.0061 | Al-Chokhachy and Budy 2008 (length to fecundity equation) |
| Egg Mortality (StDev) | 60 (2.5) | Calibrated to match empirical age distribution - Seattle City Light (unpublished data) |
| Egg Female Percentage | 50 | Fisher 1930 |
| Length at infinity (L∞) | 1134 | Zymonas 2006 |
| Growth rate (R0) | 0.75 | Zymonas 2006 |
| Growth Temp Max (˚C) | 12 | Nathan et al. 2019 |
| Age at length 0 (t_0_) | -0.2 | Nathan et al. 2019 |
| Packing Algorithm | -0.6821 | Landguth et al. 2017 |
